# Gamma-Irradiation-Prepared Low Molecular Weight Hyaluronic Acid Promotes Skin Wound Healing

**DOI:** 10.3390/polym11071214

**Published:** 2019-07-19

**Authors:** Yu-Chih Huang, Kuen-Yu Huang, Wei-Zhen Lew, Kang-Hsin Fan, Wei-Jen Chang, Haw-Ming Huang

**Affiliations:** 1 School of Dentistry, College of Oral Medicine, Taipei Medical University, Taipei 11031, Taiwan; 2 Dental Department, En Chu Kong Hospital, New Taipei City 23741, Taiwan; 3 Graduate Institute of Biomedical Optomechatronics, College of Biomedical Engineering, Taipei 11031, Taiwan; 4 Research Center of Biomedical Device, Medical University, Taipei 11031, Taiwan

**Keywords:** low molecular weight, hyaluronic acid, wound healing, gamma ray, membrane

## Abstract

In this study, we prepared low-molecular-weight hyaluronic acid (LMWHA) powder by γ-irradiation. The chemical and physical properties of γ-irradiated LMWHA and the in vitro cellular growth experiments with γ-irradiated LMWHA were analyzed. Then, hyaluronic acid exposed to 20 kGy of γ-irradiation was used to fabricate a carboxymethyl cellulose (CMC)/LMWHA fabric for wound dressing. Our results showed that γ-irradiated LMWHA demonstrated a significant alteration in carbon–oxygen double bonding and can be detected using nuclear magnetic resonance and ultraviolet (UV)-visible (Vis) spectra. The γ-irradiated LMWHA exhibited strain rate-dependent Newton/non-Newton fluid biphasic viscosity. The viability of L929 skin fibroblasts improved upon co-culture with γ-irradiated LMWHA. In the in vivo animal experiments, skin wounds covered with dressings prepared by γ-irradiation revealed acceleration of wound healing after two days of healing. The results suggest that γ-irradiated LMWHA could be a potential source for the promotion of skin wound healing.

## 1. Introduction

Wound healing is a series of processes that involves the control of inflammation, cell migration and new tissue remodeling [1,2]. It is reported that a material with anti-inflammatory, antimicrobial and antioxidant properties that promotes cell migration can serve as a potential solution for treating skin and soft-tissue wounds [3]. 

Hyaluronic acid (HA) is a biopolymer found mainly in the extracellular space [4] and joints [5,6]. The primary physiological function of HA is its buffering action, which is due to its excellent viscoelastic properties after water absorption [7]. Thus, traditionally, HA was reportedly used as a medical material for retaining skin moisture and for osteoarthritis therapy [8]. Recently, studies have shown that HA exhibits anti-inflammatory and antibacterial activities [9,10]. In addition, since free radicals can break down hyaluronic acid into smaller fragments in damaged tissues, it also has the antioxidant function of scavenging free radicals. Furthermore, it is well known that HA also can be a scaffold during tissue repair to provide cell climbing and migration opportunities [11]. With these useful functions, HA is reported to accelerate the process of wound healing [10,12,13].

Recently, many studies have investigated the association between the molecular weight of HA and its physiological functions [14,15]. In the initial stage of wound healing, high-molecular-weight HA (HMWHA) (~2000 kDa) accumulates in the extracellular matrix and binds to fibrinogen to form a clot. Thereafter, in the inflammatory stage, HMWHA is broken down into low-molecular-weight hyaluronic acid (LMWHA) (80–800 kDa) by hyaluronidase for subsequent use in healing [16,17,18]. At this stage, LMWHA is reported to participate in the inflammatory response, involving macrophage activation and chemokine expression [11]. D’Agostino et al. (2015) performed an in vitro study and concluded that LMWHA accelerated wound repair because it inhibited fibroblast differentiation and collagen deposition at this early stage [11]. These effects allow macrophages to move to the wound site to phagocytose debris and clean infectious matter [16]. Several investigations also found that LMWHA prevented oxygen free radical damage to granulation tissue [19] and increased the self-defense of skin epithelium by inducing various skin-repair-related genes [20] during the wound healing process.

It is proposed that although HMWHA is used in various medical sciences, LMWHA may provide potential beneficial effects for wound healing. However, until now, the preparation of HA with a specific molecular weight has been a complex work that is not easy to control [21]. To fabricate LMWHA efficiently, several scholars used physical (ultrasound, ozone, electron beam, γ-irradiation and thermal treatment) and chemical methods (enzyme and acid degradation) to break the primary bond of HMWHA [21,22,23]. Among these methods, γ-irradiation is reported to reduce the molecular weight of HA without structural alteration of the polymer [21,23,24,25].

It was reported that, even though the main structure of HA fragments remained intact, the water-absorbing ability was changed due to the molecular weight reduction of the polymer [23,24]. However, the in vivo evidence supporting the efficacy of using LMWHA to fabricate a wound dressing membrane is still limited. Accordingly, the purpose of this study was to prepare LMWHA powder by γ-irradiation. The prepared LMWHA was used as a material to fabricate a hydrogel dressing membrane. We hypothesized that the γ-irradiated powder could be a useful material for fabricating a hydrogel membrane for skin wound dressing.

## 2. Materials and Methods

### 2.1. Preparation of Low-Molecular-Weight Hyaluronic Acid

The HA used in this study (molecular weight (MW) 3000 kDa) was purchased from Cheng-Yi Chemical Industry Co. Ltd. (Taipei, Taiwan). Before the experiments, the HA powder, stored in tightly capped tubes, was irradiated using a cobalt-60 irradiator (Point Source, AECL, IR-79, Nordion, Canada) at 22 °C, with a dose rate of 1 kGy/h at the sample location. The γ-irradiated HA powder was divided into four groups. The first two HA groups were γ-irradiated with a dose of 20 kGy (20 h exposure) once (HA20I) and twice (HA20II), respectively. The third and fourth LMWHA groups were exposed to γ-irradiation at doses of 40 kGy (HA40) and 60 kGy (HA60) for 20 h, respectively. The unexposed HMWHA powder served as the control group (HA0). The irradiation dose was confirmed using alanine pellet dosimeters (FWT-50, Far West Technology, Inc., Goleta, CA, USA).

The molecular weights of the HA with and without γ-irradiation were measured by gel permeation chromatography. In this study, each group of HA powder was formulated into a 10 mg/mL HA solution in 0.1 M NaCl, and then 200 μL of the sample was injected into a separation module (Series 200, Perkin Elmer, Waltham, MA, USA) equipped with a chromatography column (SB-806M HQ, Shodex, Kanagawa, Japan). A refractive index (RI) detector (Series 200, Perkin Elmer, Waltham, MA, USA) was used to detect the signals. The mobile phase was 0.1 M sodium nitrate (purity: 99.9%, Merck KGaA, Darmstadt, Germany). The flow rate was 0.5 mL/min, and the analyses were performed at 25 °C. The calibration was achieved using a standard kit (Pullulan ReadyCal Kits, PSS Polymer Standards Service, Mainz, Germany). The gel permeation chromatography data were collected and analyzed using commercially available software (ChromManager 5.8, ABDC WorkShop, Taichung, Taiwan). The dispersity of each sample was obtained by calculating the ratio between weight average molecular weight (*M*_w_) and the number average molecular weight (*M*_n_) for different samples.

### 2.2. Chemical Property Analysis

The ^13^C nuclear magnetic resonance (NMR) spectra of the γ-ray treated and untreated HA were recorded at 27 °C on a 500 MHz NMR spectrometer (DRX500 Avance, Bruker BioSpin GmbH, Rheinstetten, Germany). D2O (Sigma-Aldrich, St. Louis, MO, USA) was used as the solvent in all the NMR experiments. Fourier-transform infrared (FT-IR) spectra of the samples were detected using an infrared spectrophotometer (Spectrum one, Perkin Elmer, Waltham, MA, USA). Before tests, the γ-ray treated and untreated HA powders were mixed with KBr (Sigma-Aldrich, St. Louis, MI, USA) and compressed into disks. The wavelength range was set at 650–4000 cm^−1^. Transmission mode spectra were obtained from 24 scans. To detect the UV-Vis absorption spectra of the γ-irradiated HA, samples were diluted in distilled water to a concentration of 0.2% (mg/mL). UV-Vis spectra were measured using a CT-2400 Spectrophotometer (Great Tide Instrument Co., Ltd., Taipei, Taiwan) at a wavelength range of 200 nm to 500 nm. During detection, distilled water was used as a reference.

### 2.3. Physical Property Detection

To determine the pH of the variously irradiated HA samples, the samples were diluted 1:500 in purified water and stirred for 12 h. The pH of the samples was measured with a pH meter at room temperature (Model 6173, JENCO Quality Instruments, San Diego, CA, USA) equipped with a pH electrode (HI1413, Hanna Instruments, Inc., Woonsocket, RI, USA). Before the tests, the pH meter was calibrated with pH 7 and pH 4 buffers. The rheological characteristic of the tested HA samples (prepared to 2% mg/mL solution) was measured using an oscillatory rheometer at 25 °C (Rheostress 1, Haake, Karlsruhe, Germany). The frequency range and shear ratio were set at 0.1–100 Hz. The dynamic viscosity (η*) of the tested HA was recorded as a function of the strain rate.

The moisture absorption test method was modified from that of a previous study [7]. The irradiated HA powders were dried in an oven for 24 h before testing the moisture absorption properties. Then, 0.1 g of dried HA sample was put in a 3.5 cm culture plate and placed in an incubator (REVCO RCO3000T, Thermo Fisher Scientific, Waltham, MA, USA) at a temperature of 37 °C and relative humidity of 95%. Samples were weighted every 24 h. The water absorption capacity was expressed by the change in weight of the material after the moisture had been absorbed.

### 2.4. In Vitro Cell Viability Experiments

A cell viability assay was performed to test the effect of the γ-irradiated HA samples on the viability of skin cells. The skin fibroblast cell line L929 (American Type Culture Collection, ATCC, no. CCL-1) was used for this in vitro cell analysis. The cells were seeded in 24-well plates at a concentration of 2 × 10^4^ cells/mL and were maintained in Dulbecco’s modified Eagle medium supplemented with L-glutamine and 10% fetal bovine serum (DMEM, Gibco, Grand Island, NY, USA). The cells were cultured in an incubator in an environment of 5% CO2 at 37 °C and 100% humidity. The viability of the L929 cells co-cultured with 0.1% γ-irradiated HA for six days was detected using the tetrazolium salt method (MTT, Sigma-Aldrich, St. Louis, MO, USA). Briefly, after the test cells were incubated with tetrazolium salt for 4 h, 500 μL dimethyl sulfoxide (DMSO, Sigma-Aldrich) was added and incubated overnight to solubilize the formazan dye. The optical density was determined using a microplate reader (EZ Read 400, Biochrom, Holliston, MA, USA) at a wavelength of 570/690 nm.

### 2.5. In Vivo Wound Healing Tests

#### 2.5.1. Carboxymethyl Cellulose (CMC)/LMWHA Dressing Fabrication

A CMC/LMWHA hydrogel was prepared on a nonwoven fabric as described in a previous study [26] to test the wound healing effect of the prepared LMWHA. The 15 mL of CMC (10 mg/mL) and the 15 mL of LMWHA (30 mg/mL) were mixed with a magnetic stirrer. Then, the mixture was moved to the surface of a nonwoven fabric (3 cm × 3 cm). The dressings were then put into an oven at 37 °C for 3 h to form dried hydrogel dressings. In this study, due to the chemical and physical experiments and the policy to reduce animal use based on the Helsinki Declaration, only the HA20I sample was used to fabricate the dressing for use in the animal study. Dressings prepared with CMC only were used in the control group.

#### 2.5.2. In Vivo Wound Healing Experiment

Eight healthy male Sprague Dawley rats weighing 210 to 290 g were used to assess the effects of LMWHA on wound healing. The rats were obtained from the Laboratory Animal Center at the National Applied Research Laboratories (Hsinchu, Taiwan). They were kept in hygienic cages and maintained with a 12 h light/dark cycle. The study protocol and procedure were reviewed and approved by the Institution Animal Care and Use Committee (IACUC Approval No. L10708), and all efforts were made to minimize the number of rats and suffering to produce reliable scientific data.

Before experimentation, the rats’ backs were shaved (5 cm × 5 cm) with an electric animal shaver, and 75% alcohol was used to avoid infection. The rats were anesthetized with 5% isoflurane in an anesthesia induction chamber. One linear incision wound with an area of 2 cm × 2 cm was made on the shaved area using sterile scissors. The SD rats were randomly divided into two groups, with four rats each in experimental and control group. For the experimental group, the wound sites of the rats were covered with the dressing prepared with LMWHA (HA20I). The HA-free dressing was applied to the wounds of the control animals. The covered dressings were replaced every two days during the 12 day experimental period. The rats were housed individually and kept at an environmental temperature of 21 °C and a humidity of 60 to 70% during the entire experimental period. The wound of each rat was photographed every two days with a digital camera. The recorded wound areas were measured using ImageJ software (National Institutes of Health, Bethesda, Rockville, MD, USA). The wound size was expressed as a percentage reduction of the original wound size.

### 2.6. Statistical Analysis

For cell viability and animal tests, mean values and standard deviations of each measurement were recorded. One-way analysis of variance (ANOVA) with Tukey’s post hoc and Student *t*-tests (SPSS Inc., Chicago, IL, USA) were performed to evaluate the changes between the samples and controls, for cell and animal experiments, respectively. A *p*-value lower than 0.05 was considered statistically significant.

## 3. Results and Discussion

As shown in Table 1, the molecular weights of the HA samples exposed to γ-irradiation decreased significantly in a dose-dependent manner. The molecular weight was 232.4 kDa when 20 kGy γ-radiation was applied. This value decreased to 141.8 kDa and 59.5 kDa when 40 kGy and 60 kGy γ-radiation were used, respectively. This phenomenon was similar to the results of a previous HA powder experiment [23]. However, our data were much lower than the findings of Kim et al. (2008), who used 50 kGy γ-irradiated HA dissolved in distilled water, for which the molecular weight decreased to 6.5 kDa [24]. This extreme decrease is because hydrogen and hydroxide radicals formed during the irradiation of the water, breaking the molecular chain of the HA molecules [24]. As mentioned above, there are several methods to reduce the molecular weight of HA. Among these methods, enzymatic and chemical methods are relatively uncontrollable. LMWHA prepared by these means is reported to show a broader molecular weight distribution compared to that prepared by physical techniques (Kim et al., 2008). For definition, the HA20II and HA40 samples received the same dose of γ-radiation. However, from Table 1, we found that the molecular weight and pH values of HA20II were lower than those of HA40. This may be due to the fact that the position and exposed direction of the HA20II sample was changed at the time interval between the two exposures. This procedure makes the samples received a more homogeneous γ-radiation and leads to a more serious breakdown of their molecular chains. In the present study, the polydispersity (*M*_w_/*M*_n_) decreased along with the γ-ray dose. This value reduced from 386.2 to 3.7 when 20 kGy γ-radiation was used. Since the polydispersity of a polymer is an important parameter related to degradation conditions and molecular weight distribution, our results confirm the conclusions of previous studies showing that LMWHA manufactured by γ-irradiation degrades the HMWHA powder more randomly [23,24] and makes the material more homogeneous when dissolved in water. 

**Figure 1:**
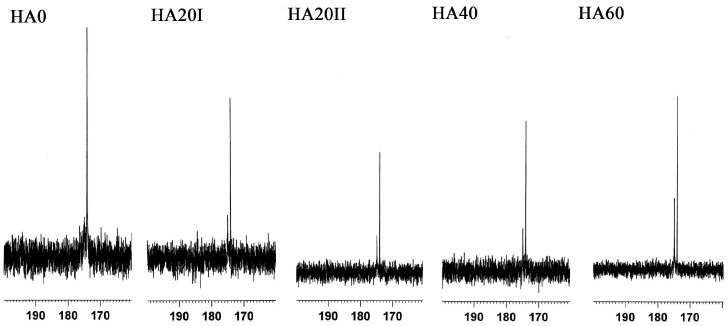
^13^C NMR spectra of γ-ray treated and untreated hyaluronic acid (HA) samples at chemical shifts between 160 and 190 ppm.

**Figure 2:**
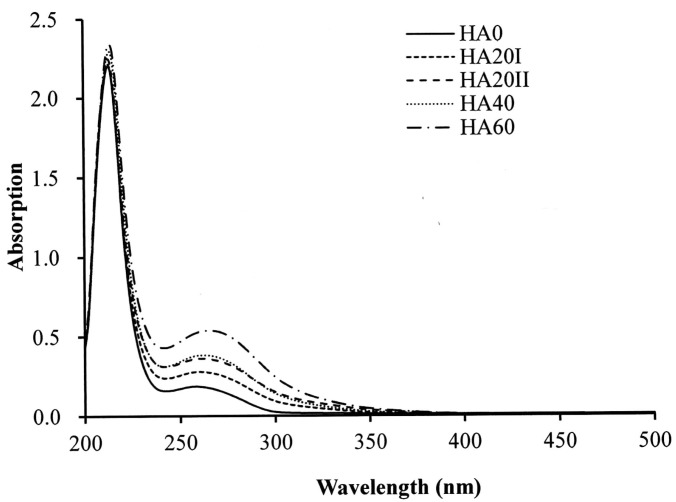
UV-Vis spectra of HA irradiated with various doses of γ-radiation. HA20I and HA20II are HA samples that received 20 kGy γ-irradiation once or twice, respectively. HA40 and HA60 represent the HA samples that received 40 kGy and 60 kGy γ-irradiation, respectively.

The samples were analyzed by FT-IR, ^13^C NMR and UV-Vis spectroscopy to confirm the LMWHA structural changes due to γ-irradiation. For the ^13^C NMR analysis, the major difference between the untreated HMWHA and γ-irradiated LMWHA can be found at chemical shifts of 171 and 175 ppm (Figure 1). According to previous reports, these peaks are due to carbon–oxygen double bonds (C=O). The 171 and 175 ppm were carboxylate carbon and acetamido carbonyl carbon, respectively [14,27,28]. When the HA samples were exposed to γ-rays, the peak ratio of 175/171 ppm markedly increased. The analysis of UV-Vis spectra (Figure 2) confirmed previous findings [23,24] that γ-irradiation increases the absorbance at 265 nm. A prior report on the effects of γ-irradiation on alginates using 60Co in the dosage range of 20 to 500 kGy indicated that the absorbance at 265 nm is due to the double bond of HA formed after the degradation of the main chain of the polymer. This effect may be attributed to a hydrogen abstraction reaction after degradation [29]. That is, the γ-irradiation of LMWHA significantly changes the chemical structure of the HA associated with carbon–oxygen double bonds. Interestingly, not all degradation methods have the same effect of increasing C=O bonding. For example, HA treated with ultrasound, hydrogen peroxide and ozone showed no apparent changes in NMR and UV-Vis spectra [21,25]. 

**Figure 3:**
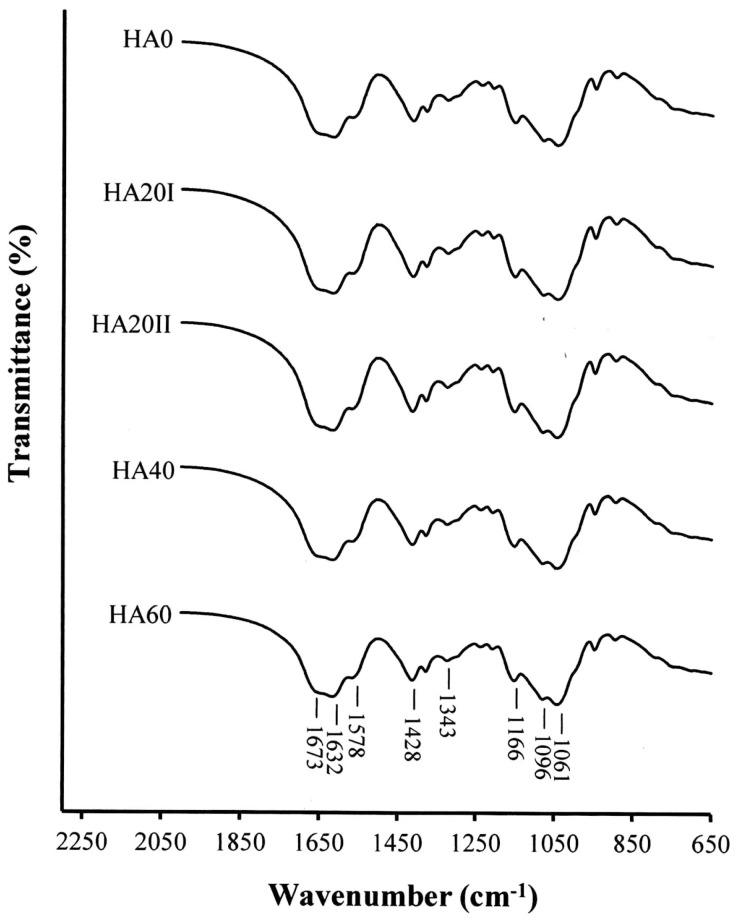
FTIR spectra of high-molecular-weight hyaluronic acid (HMWHA) and low-molecular-weight hyaluronic acid (LMWHA) treated with various doses of γ-ray irradiation.

The FT-IR spectra of LMWHA degraded from various doses of γ-irradiation are shown in Figure 3. According to previous reports [23,24,25], the absorption bands at 1061–1166 cm^−1^ are characteristic f carbohydrates. The band at 1673 cm^−1^ is associated with carbon–oxygen double bonds (C=O). The bands at 1632 cm^−1^, 1578 cm^−1^ and 1320 cm^−1^ correspond to amides. No substantial change was found when comparing the FT-IR spectra of the HMWHA (HA0) to the γ-irradiated LMWHA samples. This phenomenon differs from that of the 13C NMR and UV-Vis tests. This result may be due to the carbon–oxygen double bond-associated band (1673 cm^−1^), which already exists in untreated HMWHA and can overlap with other bands, making it hard to distinguish the molecular size of the HA.

**Figure 4:**
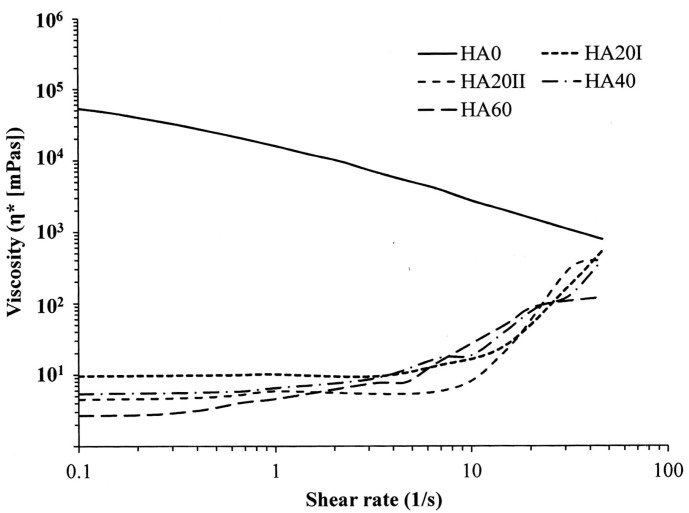
Dynamic viscosity dependencies of applied shear rates of the HMWHA and LMWHA samples.

We confirmed the conclusion of previous studies that LMWHA prepared by γ-irradiation preserves its fundamental structure, but with the formation of large amounts of the carbonyl group due to the depolymerization process [30]. This depolymerization of HA results in decreased pH values and the decreased viscosity of HA, as shown in Table 1 and Figure 4 [24,28]. In Figure 4, untreated HMWHA shows a typical viscosity pattern. The dynamic viscosity of HMWHA depends on the shear rate as a non-Newtonian liquid [31]. However, the dynamic viscosity of γ-irradiated HA markedly decreased independently with the applied shear rate when the rate was less than 10 s^−1^. That is, at this status, the γ-irradiated HA showed a Newtonian liquid viscosity behavior. This effect can be attributed to the depolymerization process due to γ-irradiation, which results in the collapse of the macromolecular coils [31]. Interestingly, when the strain rate was larger than 10 s^−1^, the γ-irradiated HA demonstrated a shear-thickening characteristic. The viscosity of the prepared LMWHA increased with increasing strain rate. This “strain-hardening” property suggests that the LMWHA fluids provide a shock-damping function and protective effect when a sudden high-load impact is applied [32].

**Figure 5:**
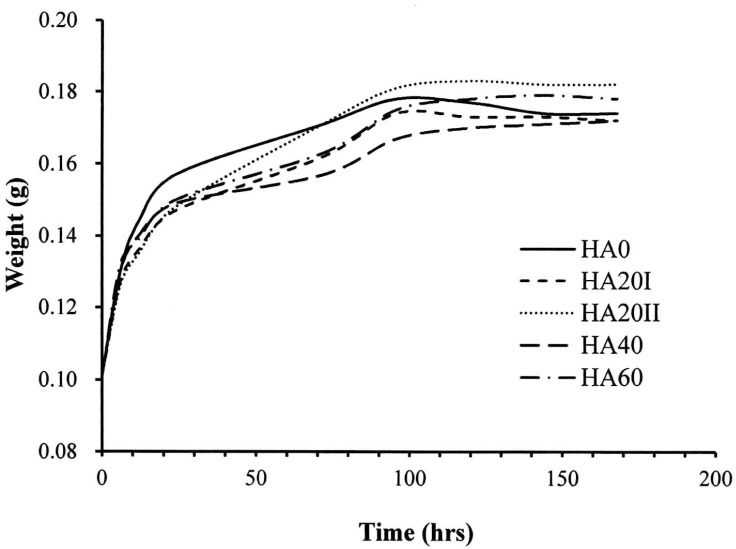
Effect of γ-irradiation on the water-absorbing ability of LMWHA over time.

It is well known that the primary function of HA is to hold water and retain a balance of moisture [33]. The critical concern of using LMWHA for wound dressing is the water-absorbing ability, which is reduced when the polymer is depolymerized to small MW fragments [23,24]. In this study, we found that the γ-irradiation reduced the water-absorbing ability when the material came in contact with water (Figure 5). However, after 15 h of experimentation, the water-absorbing abilities of all the HA samples dramatically increased and then reached a plateau at 96 h. At this time, the weight of the water-absorbed by HMWHA was almost the same as that of the HA20I sample. This phenomenon may be due to γ-irradiation and would not affect the water-holding related chemical structure. Accordingly, pre-immersion of the γ-irradiated HA in water for a certain time resolved the problem of water-absorbing ability reduction when the LMWHA was considered for use.

**Figure 6:**
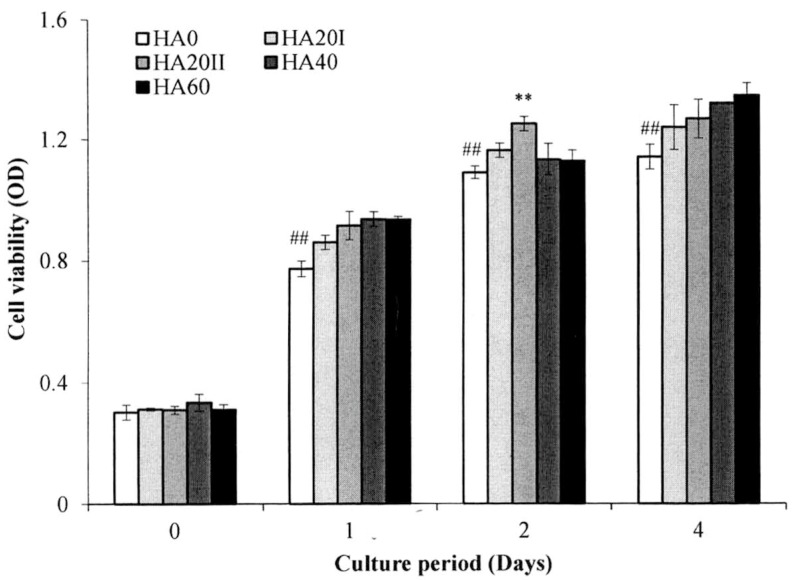
Cell viability tests for the HMWHA and LMWHA irradiated with various dosages of γ-radiation. The data are presented as mean ± SD (*n* = 84). ** and ## denote significant differences (*p* < 0.05).

The cell viability of the HA samples was evaluated using the MTT assay (Figure 6) in L929 fibroblasts. The cells cultured with HMWHA and LMWHA showed typical growth curves. The L929 fibroblasts exhibited no cytotoxicity. During the four-day experimental period, cells cultured with γ-irradiated HA exhibited significantly higher viability compared to the cells cultured with untreated HMWHA (*p* < 0.05). The result that γ-irradiated LMWHA increases skin cell proliferation becomes a crucial property for their application in wound healing [34,35]. Because the γ-irradiated HA exhibited similar properties in the chemical and physical analyses and in vitro cell experimentation, only the HA20I sample was used to fabricate the dressing fabric used in the rat study in keeping with the policy of animal reduction based on the Helsinki Declaration.

**Figure 7:**
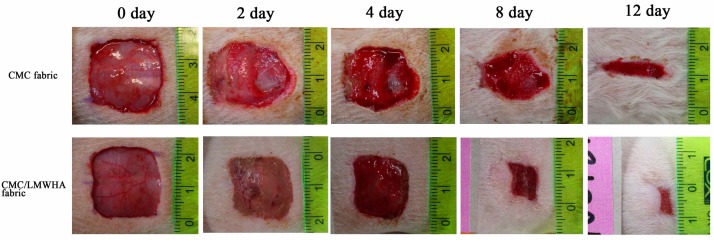
Photographs of the wound in rats after skin excision on days 0, 2, 4, 8 and 12.

**Figure 8:**
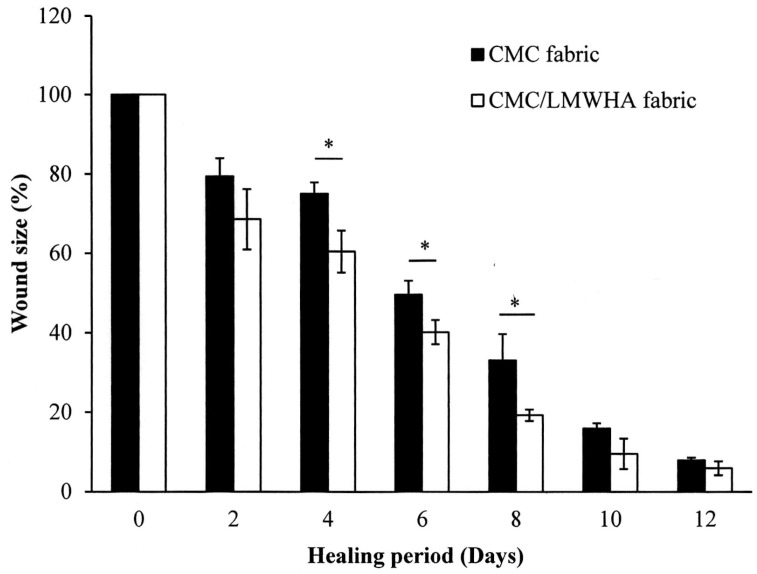
Photographs of the wound in rats after skin excision on days 0, 2, 4, 8 and 12.

The CMC/HA fabric was fabricated for wound dressing in this study. Previous studies demonstrated that CMC/HA is nontoxic, nonmutagenic, nonimmunogenic, nonirritating, nonpyrogenic [36] and did not induce an inflammatory cytokine response [37]. In addition, the fabrication of such a CMC/HA hydrogel does not require chemical additives or an energy source [26]. The in vivo wound healing activity of the CMC/LMWHA dressing is shown in Figure 7. Two days after the skin excision, the healing process inside the epidermis was better in the wound covered with CMC/LMWHA fabric compared to the CMC only fabric. The CMC/LMWHA fabric-covered wound showed accelerated healing and lower secretions than in the CMC fabric-only group. The quantitative results demonstrated that the wounds covered with CMC/LMWHA fabric (60.42 ± 5.29%) resulted in a statistically significant reduction (*p* < 0.05) in the wound area when compared to that of the wound covered with CMC fabric (75.07 ± 2.79%) (Figure 8). This statistically significant reduction in wound size was observed on days 4–8 of the experimental period. On day 8, compared to the control group, the wounds covered with CMC/LMWHA fabric became dark brown, dry and smaller than the wounds covered with the CMC fabric (Figure 7). The wound size of the CMC/LMWHA fabric group decreased significantly to 19.23 ± 1.45% (Figure 8), which is almost half that of the wounds covered with CMC fabric (33.12 ± 6.54%) (*p* < 0.05). It is known that antimicrobial activity is an essential requirement for evaluating a wound dressing material [3]. Since the growth of wound-related bacteria requires a neutral pH environment, an acidic environment is not conducive to the growth of bacteria [38,39]. Thus, the reduction in pH value due to HA depolymerization (Table 1) results in an acidic environment, which may be the reason leading to the promotion of skin cell proliferation [40] and collagen reorganization, with the resulting acceleration of wound healing [38,41] as showed in Figure 7 and Figure 8. According to these results, we confirmed that LMWHA promotes wound healing and acts as an excellent wound dressing material for medical application.

## 4. Conclusions

The LMWHA prepared by γ-irradiation shows strain rate-dependent Newton/non-Newton fluid biphasic viscosity. The water absorption ability of γ-ray-prepared LMWHA is reserved when used for long-term medical application. In addition, the LMWHA prepared by γ-irradiation can be an excellent wound dressing material for medical applications.

## Figures and Tables

**Table 1:** Molecular weights, dispersity and pH values of LMWHA irradiated with various doses of γ-irradiation.

Sample	Molecular Weight (kDa)	Dispersity (Mw/Mn)	pH
HA0	2983.7	386.2	6.76
HA20I	232.4	3.7	5.95
HA20II	99.2	3.4	5.49
HA40	141.8	3.2	5.54
HA60	59.5	2.5	5.2

## References

[1] Monsuur H.N., Boink M.A., Weijers E.M., Roffel S., Breetveld M., Gefen A., van den Broek L.J., Gibbs S. (2016). Methods to study differences in cell mobility during skin wound healing in vitro. J. Biomech..

[2] Lin T.Z., Zhong L., Santiago J.L. (2018). Anti-inflammatory and skin barrier repair effects of topical application of some plant oils. Int. J. Mol. Sci..

[3] Wang Y.F., Que H.F., Wang Y.J., Cui X.J. (2014). Chinese herbal medicines for treating skin and soft-tissue infections. Cochrane Database Syst. Rev..

[4] Korn P., Schulz M.C., Hintze V., Range U., Mai R., Eckelt U., Schnabelrauch M., Möller S., Becher J., Scharnweber D. (2014). Chondroitin sulfate and sulfated hyaluronan-containing collagen coatings of titanium implants influence peri-implant bone formation in a minipig model. J. Biomed. Mater. Res. A.

[5] Correia C.R., Moreira-Teixeira L.S., Moroni L., Reis R.L., van Blitterswijk C.A., Karperien M., Mano J.F. (2011). Chitosan scaffolds containing hyaluronic acid for cartilage tissue engineering. Tissue Eng. Part C Methods.

[6] Dahiya P., Kamal R. (2013). Hyaluronic acid: A boon in periodontal therapy. N. Am. J. Med. Sci..

[7] Zhang W., Mu H., Zhang A., Cui G., Chen H., Duan J., Wang S. (2013). A decrease in moisture absorption–retention capacity of N-deacetylation of hyaluronic acid. Glycoconj. J..

[8] Kablik J., Monheit G.D., Yu L., Chang G., Gershkovich J. (2009). Comparative physical properties of hyaluronic acid dermal fillers. Dermatol. Surg..

[9] Jentsch H., Pomowski R., Kundt G., Göcke R. (2003). Treatment of gingivitis with hyaluronan. J. Clin. Periodontol..

[10] Frenkel J.S. (2014). The role of hyaluronan in wound healing. Int. Wound J..

[11] D’Agostino A., Stellavato A., Busico T., Papa T., Tirino V., Papaccio G., La Gatta A., De Rosa M., Schiraldi C. (2015). In vitro analysis of the effects on wound healing of high- and low-molecular weight chains of hyaluronan and their hybrid H-HA/L-HA complexes. BMC Cell Biol..

[12] Voigt J., Driver V.R. (2012). Hyaluronic acid derivatives and their healing effect on burns, epithelial surgical wounds, and chronic wounds: A systematic review and meta-analysis of randomized controlled trials. Wound Repair Regen..

[13] Neuman M.G., Nanau R.M., Oruña-Sanchez L., Coto G. (2015). Hyaluronic acid and wound healing. J. Pharm. Pharm. Sci..

[14] Cowman M.K., Hittner D.M., Feder-Davis J. (1996). ^13^C-NMR studies of hyaluronan: Conformational sensitivity to varied environments. Macromolecules.

[15] Ke C., Sun L., Qiao D., Wang D., Zeng X. (2011). Antioxidant activity of low molecular weight hyaluronic acid. Food Chem. Toxicol..

[16] Maharjan A.S., Pilling D., Gomer R.H. (2011). High and low molecular weight hyaluronic acid differentially regulate human fibrocyte differentiation. PLoS ONE.

[17] Rayahin J.E., Buhrman R.S., Zhang Y., Koh T.J., Gemeinhart R.A. (2015). High and low molecular weight hyaluronic acid differentially influence macrophage activation. ACS Biomater. Sci. Eng..

[18] Kavasi R.M., Berdiaki A., Spyridaki I., Corsini E., Tsatsakis A., Tzanakakis G., Nikitovic D. (2017). HA metabolism in skin homeostasis and inflammatory disease. Food Chem. Toxicol..

[19] Trabucchi E., Pallotta S., Morini M., Corsi F., Franceschini R., Casiraghi A., Pravettoni A., Foschi D., Minghetti P. (2002). Low molecular weight hyaluronic acid prevents oxygen free radical damage to granulation tissue during wound healing. Int. J. Tissue React..

[20] Gariboldi S., Palazzo M., Zanobbio L., Selleri S., Sommariva M., Sfondrini L., Cavicchini S., Balsari A., Rumio C. (2008). Low molecular weight hyaluronic acid increases the self-defense of skin epithelium by induction of-defensin 2 via TLR2 and TLR4. J. Immunol..

[21] Chen H., Qin J., Hu Y. (2019). Efficient degradation of high-molecular-weight hyaluronic acid by a combination of ultrasound, hydrogen peroxide, and copper ion. Molecules.

[22] Hokputsa S., Jumel K., Alexander C., Harding S.E. (2003). A comparison of molecular mass determination of hyaluronic acid using SEC/MALLS and sedimentation equilibrium. Eur. Biophys. J..

[23] Choi J., Kim J.K., Kim J.H., Kweon D.K., Lee J.W. (2010). Degradation of hyaluronic acid powder by electron beam irradiation, gamma ray irradiation, microwave irradiation and thermal treatment: A comparative study. Carbohydr. Polym..

[24] Kim J.K., Sung N.Y., Srinivasan P., Choi J.I., Kim S.K., Oh J.M., Kim J.H., Song B.S., Park H.J., Byun M.W. (2008). Effect of gamma irradiated hyaluronic acid on acetaminophen induced acute hepatotoxicity. Chem. Biol. Interact..

[25] Yue W. (2012). Preparation of low-molecular-weight hyaluronic acid by ozone treatment. Carbohydr. Polym..

[26] Huang Y.C., Huang K.U., Yang B.Y., Ko C.H., Huang H.M. (2016). Fabrication of novel hydrogel with berberine-enriched carboxymethylcellulose and hyaluronic acid as an anti-inflammatory barrier membrane. BioMed Res. Int..

[27] Scott J.E., Heatley F. (1999). Hyaluronan forms specific stable tertiary structures in aqueous solution: A 13C NMR study. Proc. Natl. Acad. Sci. USA.

[28] Scott J.E., Heatley F. (2002). Biological properties of hyaluronan in aqueous solution are controlled and sequestered by reversible tertiary structures, defined by NMR Spectroscopy. Biomacromolecules.

[29] Nagasawa N., Mitomo M., Yoshii F., Kume T. (2000). Radiation-induced degradation of sodium alginate. Polym. Degrad. Stab..

[30] Gura E., Huckel M., Muller P.J. (1998). Specific degradation of hyaluronic acid and its rheological properties. Polym. Degrad. Stab..

[31] Lapčík L., Benešová K., Lapčík L., De Smedt S., Lapčíková B. (2010). Chemical modification of hyaluronic acid: Alkylation. Int. J. Polym. Anal. Charact..

[32] Haward S.J., Jaishankar A., Oliveira M.S.N., Alves M.A., McKinley G.H. (2013). Extensional flow of hyaluronic acid solutions in an optimized microfluidic cross-slot device. Biomicrofluidics.

[33] Necas J., Bartosikova L., Brauner P., Kolar J. (2008). Hyaluronic acid (hyaluronan): A review. Vet. Med. Czech.

[34] Lönnroth E.C. (2005). Toxicity of medical glove materials: A pilot study. Int. J. Occup. Saf. Ergon..

[35] Eskandarinia A., Kefayat A., Rafienia M., Agheb M., Navid S., Ebrahimpour K. (2019). Cornstarch-based wound dressing incorporated with hyaluronic acid and propolis: In vitroand in vivo studies. Carbohydr. Polym..

[36] Burns J.W., Colt M.J., Burgees L.S., Skinner K.C. (1997). Preclinical evaluation of Seprafilm bioresorbable membrane. Eur. J. Surg. Suppl..

[37] Uchida K., Otake M., Inoue Y., Koike K., Matsushita K., Tanaka K., Inoue Y., Mohri Y., Kusunoki M. (2001). Bacteriostatic effects of hyaluronan-based bioresorbable membrane. Surg. Sci..

[38] Rekik D.M., Khedir S.B., Moalla K.K., Kammoun N.G., Rebai T., Sahnoun Z. (2016). Evaluation of wound healing properties of grape seed, sesame, and fenugreek oils. Evid. Based Complement. Altern Med..

[39] Saeidnia S., Manayi A., Gohari A.R., Abdollahi M. (2014). The story of beta-sitosterol—A review. Eur. J. Med. Plants.

[40] Chen C.C., Nien C.J., Chen L.G., Huang K.Y., Chang W.J., Huang H.M. (2019). Effects of Sapindus mukorossi seed oil on skin wound healing: In vivo and in vitro testing. Int. J. Mol. Sci..

[41] Mwipatayi B.P., Angel D., Norrish J., Hamilton M.J., Scott A., Sieunarine K. (2004). The use of honey in chronic leg ulcers: A literature review. Prim. Intent..

